# Negativity Bias in Depression and Anxiety: Examining the Psychometric Properties of a Modified Scrambled Sentences Task to Measure Interpretation Bias

**DOI:** 10.3390/bs16050705

**Published:** 2026-05-05

**Authors:** Kate Rho, Ashali Kataria, Susan A. J. Birch, Joelle LeMoult

**Affiliations:** 1 Department of Educational and Counselling Psychology, and Special Education, University of British Columbia, 2136 West Mall, Vancouver, BC V6T 1Z4, Canada; hrho01@student.ubc.ca; 2 Department of Psychology, University of British Columbia, 2136 West Mall, Vancouver, BC V6T 1Z4, Canada; ashkat25@student.ubc.ca (A.K.); jlemoult@psych.ubc.ca (J.L.)

**Keywords:** interpretation bias, depression, anxiety, negative affect, scrambled sentence task, psychopathology, cognitive bias

## Abstract

In the Scrambled Sentences Task (SST), a widely used experimental paradigm for measuring negative interpretation bias (IB), participants are shown sets of ‘scrambled’ words that can be rearranged into either negative or positive sentences. The SST is traditionally administered with time limits, cognitive load and negative mood induction. Yet, whether these procedural features are required in all contexts remains uncertain. We tested whether a modified SST (SST-M), lacking time limits, cognitive load and mood induction, can be sufficient to detect associations between IB and current psychopathology by examining validity evidence and reliability of scores. Sixty-six Canadian university students (80.3% Women) completed the SST-M and measures of anxiety, depression, negative affect, and growth mindset. Findings offer preliminary validity evidence supporting the interpretation of SST-M scores as reflecting IB in a student sample. Results yielded excellent internal consistency and expected associations with anxiety, depression, negative affect, and growth mindset. The SST-M also displayed incremental associations above and beyond demographic covariates and negative affect. Our findings suggest that IB can be reliably detected, in at least some samples, without time limits, cognitive load and mood induction. The SST-M is an easy-to-administer measure that lowers data collection barriers, providing the potential to advance research across broader populations.

## 1. Introduction

Have you ever seen someone’s face and assumed they were upset with you, only to find out they were actually just tired or lost in thought? In everyday life, we frequently encounter ambiguous situations that can be interpreted in multiple ways. Negative interpretation bias (IB) refers to a tendency to interpret ambiguous cues, stimuli, or situations in a negative manner (Lawson et al., 2002). We are all susceptible to IB as humans; however, when it becomes persistent, it is associated with psychopathology (for a review, see Hirsch et al., 2016). Following Beck’s seminal cognitive theory of depression (Beck, 1967), a wealth of research has established that IB plays a central role in the development and maintenance of depression (for a review, see Everaert et al., 2017; see Grocott et al., 2023, for longitudinal evidence). Similarly, meta-analytic evidence indicates that IB is also robustly associated with anxiety (for a review, see Würtz et al., 2026). Despite its theoretical and clinical importance, the question of how best to identify and measure IB is still a matter of considerable debate. Researchers have adopted a wide range of measurement tools to assess IB. Notably, IB has been conceptualized to occur at different levels of processing, with some interpretations occurring more deliberately and others occurring at an automatic level (for a review, see Würtz & Sanchez-Lopez, 2023). Correspondingly, assessment tools vary in the extent to which they capture deliberate versus automatic processes. There are a number of self-report or scenario-based measures designed to tap into individuals’ deliberate interpretations (Würtz & Sanchez-Lopez, 2023). For example, sentence completion tasks (e.g., S. Barton et al., 2005; S. B. Barton & Morley, 1999; Huppert et al., 2007) ask participants to finish short ambiguous sentences in ways that reveal their typical interpretive style. The Cognitive Bias Questionnaire (Krantz & Hammen, 1979) presents everyday scenarios and has respondents choose the explanation they find most likely. The Interpretation Questionnaire (Amir et al., 1999) presents ambiguous situations, and participants rank positive, neutral, and negative interpretations of them. Yet, due to the reflective nature of the methods, these assessment tools have been criticized for being vulnerable to response biases such as social desirability bias (Würtz & Sanchez-Lopez, 2023). Furthermore, for individuals with limited metacognitive awareness, these methods may fail to accurately capture underlying IB.

Experimental measures, on the other hand, such as the homophone task (Hadwin et al., 1997) and the lexical decision task (Hirsch & Mathews, 1997) have been designed to capture IB at an automatic level (Würtz & Sanchez-Lopez, 2023). These tasks present ambiguous stimuli and require participants either to identify the meaning they perceive in homophones (e.g., “die” vs. “dye”) or to quickly judge whether a letter string is a real word or a non-word in a lexical decision task (e.g., “panic” vs. “panicd”). Although these tasks address the aforementioned response bias concerns associated with self-report and scenario-based methods, these measures employ isolated words that are not explicitly framed as self-relevant, which may limit how closely they capture interpretation processes as they occur in everyday situations. Self-relevance is believed to be an important feature of an IB measure, since IB tends to be stronger in the presence of self-referential information (for a review, see Everaert et al., 2017).

One task that addresses these concerns and has accumulated substantial evidence for validity as a behavioural measure of IB is the scrambled sentences task (SST). The SST was originally developed as a measure of hostile cognition by R. E. Watson et al. (1955) and later adapted into a group-administered format by Costin (1969). It was later modified by Wenzlaff and Bates (1998) to examine patterns of cognitive vulnerability in depressive symptoms. The SST is now a widely used experimental paradigm for measuring IB associated with a number of mental health conditions (for a review, see Würtz et al., 2022). Briefly, the SST involves a series of scrambled words that participants rearrange into either a negative or positive grammatical sentence. Importantly, the SST incorporates self-referential stimuli; that is, the sentences that participants are asked to form are evaluative statements about themselves (e.g., “I am confident/disappointed in myself”). At the same time, the SST may be less influenced by response biases than self-report or scenario-based measures, given its more behavioural and indirect nature (Würtz & Sanchez-Lopez, 2023). That is, because the SST does not require participants to directly report on their biases, it may reduce reliance on meta-cognitive awareness (Würtz & Sanchez-Lopez, 2023).

Traditionally, the SST is administered with time limits and a concurrent cognitive task (e.g., memorizing a 6-digit number while engaging in the SST; Wenzlaff & Bates, 1998). These approaches were implemented with the intention of preventing, or at least minimizing, deliberate processing and eliciting automatic interpretations (Everaert et al., 2013; Lin et al., 2020; Rude et al., 2002). Furthermore, some researchers use a negative mood induction prior to the SST administration (e.g., Phillips & Hine, 2013; Sfärlea et al., 2020). This is based on the assumption that negative mood can activate latent maladaptive schemas and underlying cognitive processing patterns (Beck, 2008; Scher et al., 2005), particularly among individuals with latent vulnerability, such as those with remitted depression (Gemar et al., 2001; Lau et al., 2004) or familial risk (Kujawa et al., 2011). However, it is not implemented consistently (Würtz et al., 2022).

These procedural considerations are relevant to an ongoing debate around whether the SST measures automatic versus deliberate levels of IB. That is, as mentioned above, the SST intends to measure automatic processing of IB by incorporating time limits and concurrent cognitive load. However, a growing number of scholars have suggested that even under these conditions, the SST may still involve deliberate processes. For example, scholars have pointed out that, in the SST, participants need to actively evaluate and select between alternative sentences (i.e., positive versus negative), and participants may be influenced by demand characteristics as the purpose of the SST may be easy to infer (Everaert et al., 2017; Phillips & Hine, 2013). Accordingly, scholars have started to position SST as a measure that captures both automatic and deliberate processes (Würtz et al., 2022; Würtz & Sanchez-Lopez, 2023).

A growing body of research suggests that the necessity of these added procedures, such as time limits, cognitive load, and mood induction, may vary across samples and study contexts. For example, longer response times in a study by Viviani et al. (2018) were associated with a greater likelihood of forming negative sentences. These results challenge the assumptions that extended response time and deliberation increase the likelihood of socially desirable response patterns (i.e., forming positive sentences). Similarly, a recent systematic review and meta-analysis (Würtz et al., 2022) found that administering cognitive tasks in concert with the SST was not a significant moderator of convergent validity or internal consistency, especially among cross-sectional studies. Lastly, experimental work examining mood inductions in samples of university students has shown that changes in mood did not alter SST performance (Standage et al., 2010; see also Bisson & Sears, 2007 for evidence from other IB paradigms), consistent with the aforementioned theoretical accounts suggesting that mood-induced activation of negative IB is characteristic of at-risk individuals (Gemar et al., 2001; Lau et al., 2004). Together, these findings suggest that the experimental additions, such as time limit, cognitive load, and mood induction, which are commonly used in SST administration, may not always be necessary. Furthermore, these procedural additions may also be less appropriate for some populations, such as neurodivergent or cognitively diverse populations, for whom performance under time pressure and cognitive load may reflect differences in executive functioning rather than underlying IB (Fisher et al., 2023; McMillan et al., 2023). From an implementation perspective, using an SST with time limits and cognitive load can require specialized experimental software (e.g., E-Prime or comparable platforms; Sanchez et al., 2015; O’Connor et al., 2021) and/or in-person, lab-based administration, making them more resource-intensive and less accessible compared to self-administered online tools.

The primary objective of our current research was to examine whether a modified version of the SST (hereon termed SST-M) that lacked specific time constraints, cognitive load and negative mood induction was sufficient to find a relationship between IB and current psychopathology symptoms in a university student sample. A concurrent objective was to thereby create an IB measure that is substantially easier for researchers to administer, and more feasible to use across larger and broader populations. Building on the perspective that SST performance reflects both automatic and deliberate processes, the present study conceptualizes the SST-M as assessing the same underlying construct of IB as the traditional SST, yet under lower task constraints.

To address these objectives, we developed and tested the SST-M, a self-administered online version of the SST without time limits, cognitive load, or mood induction. Participants completed the SST-M along with well-established measures of depression, anxiety, negative affect, and growth mindset. We examined validity evidence and reliability of scores from the SST-M and tested the following hypotheses: First, we expected the SST-M to provide evidence of acceptable internal consistency reliability (H1; *α* > 0.70). Second, we examined evidence based on relations with other variables, specifically convergent validity, by testing whether higher negative SST-M scores were associated with higher levels of depression and anxiety, with correlations expected to be at least of moderate magnitude (H2; r > 0.30). Third, we evaluated evidence of incremental validity as part of relations with other variables, testing whether the SST-M would explain unique variance in depression and anxiety above and beyond negative affect (H3). The analytic plan corresponding to these hypotheses was preregistered with the Open Science Foundation (OSF; Rho et al., 2025). In addition, we examined evidence based on relations with other variables, specifically discriminant validity, by testing associations with growth mindset and negative affect, which were expected to be weaker relative to those observed for depression and anxiety.

## 2. Materials and Methods

### 2.1. Participants

A total of 81 participants were recruited via a university psychology research pool, which consists of undergraduate students who receive course credit for participating in research studies, between February 2025 and April 2025. Our final analytical sample included 66 participants (*M*_age_ [*SD*] = 20.58 [1.93], 80.3% Women). To be eligible for the study, participants had to be 18 years or older and comprehend written English. Of the 81 participants who met the inclusion criteria, two were excluded due to completing less than 85% of the survey items, and an additional four were excluded because they answered “no” to an inclusion criterion question asking whether they took this study seriously. Our inclusion criteria included passing 2 attention checks embedded in the survey to minimize concerns regarding random responding and participant fatigue. No participants who met the other inclusion criteria failed to meet this inclusion criterion. Nine participants were excluded due to incorrect completion of the SST-M task trials (i.e., including both negatively and positively valenced words in a sentence or failing to write a grammatically correct sentence). See Supplementary Materials (Table S1) for descriptive comparisons of included and excluded participants. Participants predominantly identified as Asian (57.6%) and White (28.8%), with the rest identifying as Latinx (3%) or choosing to describe their own racial identities (10.6%)^1^. See Table 1 for detailed participant demographic information. This sample would be considered an ‘unselected’ sample rather than either ‘clinical’ or ‘non-clinical’, as we did not select for individuals with current psychopathology nor make exclusions for psychopathology in the interest of maintaining a representative university student sample. The sample size was not determined a priori; nonetheless, sensitivity analyses conducted after data collection, based on a priori specified effect sizes, indicated sufficient power to detect medium-sized effects. The details of our preregistered analytic plan are available on OSF (Rho et al., 2025).

### 2.2. Materials

#### 2.2.1. Interpretation Bias (IB)

We modified the Scrambled Sentences Task (SST; Wenzlaff & Bates, 1998) in two main ways: first, we converted the SST into a completely self-administered online format; second, we removed elements argued to trigger or enhance automatic processing (i.e., time limits, cognitive load, mood induction).

As mentioned above, administration of the original SST involves a series of trials in which participants are presented with a set of ‘scrambled’ words (e.g., I failure generally a am success) that can be rearranged into either a negatively valenced (e.g., I am generally a failure) or a positively valenced grammatically correct sentence (e.g., I am generally a success). Each trial is presented sequentially (i.e., one trial per screen at a time) with a time limit (e.g., 7–12 s; Beevers & Meyer, 2008; Blanco et al., 2021; Lin et al., 2020; Sanchez et al., 2015; Sfärlea et al., 2020; Viviani et al., 2018). Prior to the first trial, participants are typically instructed to engage in a concurrent cognitive load task, such as memorizing a sequence of digits and holding them in mind during the SST task (for a review, see Würtz et al., 2022). In the SST-M, experimenter supervision, time limits, cognitive load, and mood induction were all removed. Participants completed a total of 30 trials, each consisting of a scrambled sentence (e.g., “my on I mistakes achievements focus”, “worthwhile person I worthless am a”, “laughing feel crying like I often”), with no practice block provided. Responses were entered into a text entry box, and minor spelling variations were accepted. Scoring was conducted manually. Participants were instructed at the beginning of the task to complete the task “as quickly as [they] can”, but no time limits were imposed. This modification kept the task from deviating heavily from traditional methods using time restrictions, while still allowing for easier administration.

In both the original SST and our modified version, scores are calculated by the proportion of negatively interpreted grammatically correct sentences out of the participant’s total of grammatically correct sentences. Thus, SST-M scores range from 0 to 1, with scores closer to 1 reflecting greater tendency to engage in negative IB.

#### 2.2.2. Anxiety

To mitigate issues of content heterogeneity documented across numerous established self-report anxiety measures (Wall & Lee, 2022), we administered two independent anxiety measures to reduce construct underrepresentation and measurement bias: Hospital Anxiety and Depression Scale-Anxiety (HADS-A; Zigmond & Snaith, 1983) and General Anxiety Disorder-7 (GAD-7; Spitzer et al., 2006).

Hospital Anxiety and Depression Scale-Anxiety (HADS-A). Hospital Anxiety and Depression Scale (HADS; Zigmond & Snaith, 1983) is a 14-item questionnaire consisting of two 7-item subscales—HADS-D for depression and HADS-A for anxiety. HADS-A was used in the current study to measure anxiety symptoms. Total scores range from 0 to 21, with greater scores indicating greater symptoms. In the current study, a cut-off score of 11 was used to indicate probable anxiety disorders as per a recommendation made by prior research to reduce the likelihood of false positives (Zigmond & Snaith, 1983). Previous research among university students reported evidence of strong score stability over time (test–retest reliability; r = 0.80), yet relatively weaker internal consistency (Cronbach’s alpha [*α*] = 0.72; Langvik et al., 2016). In the current sample, however, HADS-A demonstrated evidence of good internal consistency (Cronbach’s alpha [*α*] = 0.84, categorical omega [*ωC*] = 0.88).

General Anxiety Disorder-7 (GAD-7). The GAD-7 (Spitzer et al., 2006) is a 7-item questionnaire assessing the severity of generalized anxiety disorder symptoms (Spitzer et al., 2006). Total scores range from 0 to 21, with higher scores indicating greater symptoms of GAD-7. In the current study, a cut-off score of 10 or higher was used to indicate clinical levels of anxiety symptoms as per DSM V criteria (Spitzer et al., 2006). Among university students, GAD-7 has demonstrated evidence of excellent internal consistency (*α* = 0.91 − 0.93), as well as acceptable evidence based on relations to other variables, including convergent validity evidence (Byrd-Bredbenner et al., 2020). In the current sample, GAD-7 displayed evidence of excellent internal consistency (*α* = 0.92, *ωC* = 0.93), consistent with prior research.

#### 2.2.3. Depression

Consistent with the rationale for anxiety, two independent measures were administered to address content heterogeneity within widely used depression measures (Fried, 2017): the Hospital Anxiety and Depression Scale–Depression (HADS-D; Zigmond & Snaith, 1983) and the Patient Health Questionnaire (PHQ-9; Kroenke et al., 2001).

Hospital Anxiety and Depression Scale–Depression (HADS-D). HADS-D (Zigmond & Snaith, 1983) is a 7-item measure assessing depressive symptoms. Total scores range from 0 to 21, with higher scores indicating greater depressive symptoms. Although a cut-off score of 8 is commonly used for the HADS-D, in the current study, a cut-off score of 11 was used, a threshold that has been shown to produce prevalence estimates that most closely approximate clinical diagnoses of depression (Brehaut et al., 2020; see also Zigmond & Snaith, 1983). In the current sample, HADS-D displayed evidence of acceptable internal consistency (*α* = 0.79, *ωC* = 0.82).

Patient Health Questionnaire-9 (PHQ-9). The PHQ-9 is a 9-item questionnaire designed to assess symptoms of major depressive disorder based on the DSM-V criteria (Kroenke et al., 2001). Total scores range from 0 to 27, with higher scores reflecting greater symptom severity. In the current study, a cut-off score of 10 was used to indicate clinical depressive symptoms, consistent with prior research, while acknowledging that no single cut-off is uniquely optimal. Among university students, the PHQ-9 has demonstrated evidence of acceptable internal consistency, acceptable evidence based on its relation to other variables addressing convergent validity, and evidence of measurement invariance across gender, race, and ethnicity (Keum et al., 2018). In the current sample, PHQ-9 displayed evidence of good to excellent internal consistency (*α* = 0.89, *ωC* = 0.91).

#### 2.2.4. Growth Mindset

The Implicit Theory of Intelligence Scale (ITIS; Dweck et al., 1995) is a three-item questionnaire designed to assess growth mindset tendencies regarding intelligence. Growth mindset is best described as the belief that personal traits, attributes, or abilities such as intelligence or athleticism can be changed through effort, experience, or appropriate strategies (Dweck, 2006). Total scores on the ITIS are calculated by averaging the three item scores and range from 1 to 6, with higher scores representing greater growth mindset tendencies (Dweck et al., 1995). The ITIS has been shown to display evidence of excellent internal consistency (*α* = 0.94 to 0.98) and strong score stability over time (test–retest reliability; r = 0.80) among university students (for a review, see Dweck et al., 1995).

We used growth mindset tendencies to test discriminant validity. We viewed growth mindset as a good candidate for testing discriminant validity because it is related to our outcome variables, yet theoretically distinct from IB (McDonald, 1985; Roemer et al., 2021). That is, growth mindset tendencies are similarly rooted in cognitive bias and have well-documented connections with social–emotional health, such as fewer mental health issues (Hu et al., 2022; Lei et al., 2024; Tao et al., 2022). Importantly, however, growth mindset is a theoretically distinct construct from IB in that growth mindset reflects a broader belief system, whereas IB reflects a lower-level cognitive system focused on interpretations of specific ambiguous stimuli or situations. Although more proximal cognitive constructs may provide a stronger test of discriminant validity, such measures were not included in the larger project from which these data were drawn. In the current sample, ITIS displayed evidence of excellent internal consistency (*α* = 0.95, *ωC* = 0.94).

#### 2.2.5. Negative Affect

Positive and Negative Affect Schedule (PANAS; D. Watson et al., 1988) is a 20-item questionnaire consisting of two 10-item subscales—Negative Affect (NA) and Positive Affect (PA). PANAS-NA subscale includes 10 negative affect items, distressed, upset, guilty, hostile, irritable, ashamed, nervous, jittery, and afraid, and was used to measure participants’ current negative affect (D. Watson et al., 1988). Total scores range from 10 to 50, with greater scores indicating greater levels of NA. PANAS-NA has demonstrated good internal consistency (*α* = 0.85), along with evidence for construct, convergent, and discriminant validities, and measurement invariance across age, gender, and education, among a community sample of adults (Crawford & Henry, 2004). Negative affect was used in the current study for two distinct purposes. First, associations between IB and negative affect were examined as evidence of discriminant validity, given that negative affect reflects a related yet theoretically distinct construct from IB (McDonald, 1985; Roemer et al., 2021). Second, negative affect was included as a covariate in hierarchical regression analyses to examine whether SST-M scores explain unique variance of depression above and beyond general distress (i.e., evidence for incremental validity). That is, we included negative affect to test whether SST-M scores remained significantly associated with depression after accounting for general distress (i.e., negative affect), further supporting SST-M’s incremental association with depressive symptoms. In the current sample, PANAS-NA displayed good to excellent evidence of internal consistency (*α* = 0.89, *ωC* = 0.86).

#### 2.2.6. Attention Checks

Two multiple-choice attention checks were included. Attention Check #1 read “This is an attention check, please choose option ‘B’ below”. Attention Check #2 read, “This is an attention check, please choose “elephant” from the list below”.

### 2.3. Procedure

This study was administered via online questionnaires emailed to interested participants enrolled in an undergraduate Psychology course. This study was part of a larger research initiative aimed at developing and evaluating the efficacy of an online, self-paced intervention study for reducing cognitive biases and improving social-emotional well-being. It was reviewed and approved by the University of British Columbia’s Behavioural Research Ethics Board (H24-02017). Only the data and procedural components relevant to the current research aims are reported here. Notably, participants completed all of the measures in the current study prior to participating in the intervention portion of the broader research initiative. Participants provided online consent prior to completing a 45-min online survey delivered via Qualtrics with attention checks to minimize random responding and participant fatigue. The measures appeared in the following order for all participants: demographic questionnaire, the ITIS, PHQ-9, GAD-7, PANAS-NA, Attention Check # 1, SST, HADS, Attention Check #2. Participants received bonus credits toward their Psychology course for their participation in the study.

### 2.4. Analytic Plan

The analytic plan was finalized and pre-registered prior to analyses (Rho et al., 2025). All preregistered analyses were conducted as specified, and no deviations from the preregistered plan occurred except, as noted below, the addition of Steiger’s Z tests (based on a reviewer’s suggestion), the inclusion of discriminant validity analyses, and categorical omega. To evaluate evidence of internal consistency reliability of the SST-M, we pre-registered to calculate Cronbach’s alpha for the SST-M for consistency and to allow comparisons with prior literature that predominantly used Cronbach’s alpha. We also calculated categorical omega, a more robust reliability estimate for dichotomous items (Petersen, 2024). To evaluate evidence based on relations to other variables, zero-order Pearson correlations were conducted between the SST-M and depression (PHQ-9 and HADS-D) and anxiety (HADS-A and GAD-7) as convergent evidence. Hierarchical multiple regression analyses were conducted to examine incremental validity evidence, with depression (PHQ-9 and HADS-D) and anxiety (HADS-A and GAD-7) as dependent variables. Following prior literature on predictors of depression (Clark & Watson, 1991; Crawford & Henry, 2004; Salk et al., 2017), gender was entered at Step 1, negative affect was added at Step 2, and the SST-M was entered at Step 3. All analyses were conducted in R (R Core Team, 2021). In addition to the pre-registered analyses, bi-variate correlations were used to examine discriminant evidence on relations to other variables between the SST-M and negative affect (PANAS-NA) and growth mindset (ITIS). Steiger’s Z tests were conducted to compare the magnitude of the SST-M correlations with depression and anxiety measures to those with growth mindset and negative affect. For the PHQ-9 and the GAD-7, participants with ≤1 missing item on the questionnaire (*n* = 1 for each) had the missing value replaced using the prorated mean of their completed items. For the PANAS-NA, participants who missed ≥2 items (*n* = 2) were excluded from analyses involving this measure only.

## 3. Results

### 3.1. Preliminary Analyses

First, we analyzed the means and standard deviations of each measure. Descriptive statistics for study variables can be found in Table 2. On average, participants reported mild to moderate depressive symptoms on the PHQ-9 (*M* = 9.77, *SD* = 6.27) and non-clinical level of depressive symptoms on the HADS-D (*M* = 6.33, *SD* = 4.10). For anxiety, on average, participants reported mild symptoms on both the GAD-7 (*M* = 7.32, *SD* = 5.78) and HADS-A (*M* = 9.64, *SD* = 4.56). However, 42.4% of our participants scored above the clinical cut-off on the PHQ-9 and 16.7% on the HADS-D. For anxiety symptoms, 33.3% scored above the clinical cut-off on the GAD-7 and 39.4% on the HADS-A.

Next, we examined the zero-order correlations between all measures. Table 3 presents the zero-order correlations among all study variables. We tested for gender differences using independent samples t-tests. Men (*M* = 0.17) scored significantly lower than women (*M* = 0.27) on the SST-M, *t*(33.53) = −2.15, *p* = 0.039, 95% CI [−0.18, −0.01], *d* = −0.48. All other measures (ITIS, PHQ-9, GAD-7, HADS-A, HADS-D, and NA) showed no significant gender differences.

Finally, we conducted Shapiro–Wilk tests and visually inspected the histograms and Q–Q plots. Although Shapiro–Wilk tests indicated statistical non-normality for some variables, and visual inspection of histograms and Q–Q plots showed mild skewness, skewness (−0.36 to 0.91) and kurtosis (−0.13 to 0.83) values were well within accepted thresholds. No outliers were detected, and visual inspection of scatterplots confirmed linearity of the tested relationships. Given the documented robustness of Pearson correlations to mild non-normality, especially with moderate sample size (Bishara & Hittner, 2012, 2015), Pearson correlations were deemed appropriate.

Assumptions of linear regression were evaluated at the model level. Visual inspection of Q–Q plots indicated that residuals were approximately normally distributed across all models, with only minor deviations observed in the tails. Overall, the assumption of normality of residuals was deemed to be adequately met. Lastly, variance inflation factors (VIF) were examined to assess multicollinearity among predictors. All VIF values were close to 1 (range = 1.02–1.06), indicating no evidence of multicollinearity.

### 3.2. Evidence of Internal Consistency Reliability

Overall, the SST-M displayed excellent internal consistency (*α* = 0.89, *ωC* = 0.99). Corrected trial–total correlations ranged from −0.02 to 0.74. Inspection of item-level distributions indicated that four SST-M trials were highly skewed (>90% endorsement of positive-valenced sentences) with reduced variance. Categorical omega was estimated from tetrachoric correlations, which assume underlying continuous and normally distributed variables. Therefore, it is possible that the observed skew and limited variability may inflate inter-item associations, which in turn, may inflate omega coefficients. The majority of the SST-M trials (90%) displayed moderate to large associations (r ≥ 0.30) with the total score. Three trials showed lower correlations (r < 0.30). Because of the performance-based nature of the SST-M and its use of a proportion score as opposed to a sum score, these indices are interpreted descriptively rather than as criteria for item retention. We provide full item-level statistics (including item wordings, means, variances, and item–total correlations) in the Supplementary Materials (Table S2).

### 3.3. Evidence Based on Relations to Other Variables

#### 3.3.1. Convergent Evidence

Bivariate Pearson correlation analyses found that the SST-M was positively, moderately correlated with PHQ-9 (r = 0.515, *p* < 0.001) as well as with the HADS-D (r = 0.540, *p* < 0.001). Similarly, the SST-M was positively and moderately correlated with the GAD-7 (r = 0.478, *p* < 0.001) as well as the HADS-A (r = 0.544, *p* < 0.001).

#### 3.3.2. Discriminant Evidence

Bivariate Pearson correlation analyses found that the SST-M and the ITIS showed a non-significant, very small correlation (*r* = −0.08, *p* = 0.524, n.s.). Similarly, the SST-M and PANAS-NA showed a non-significant, small correlation (*r* = 0.147, *p* = 0.247, n.s.). Steiger’s Z tests were conducted to compare the strength of the SST-M correlations with depression and anxiety measures against those with growth mindset and negative affect. Across all comparisons, the SST-M demonstrated significantly stronger associations with PHQ-9, HADS-D, GAD-7, and HADS-A than with ITIS (Zs = 3.31–3.77, ps < 0.001) and PANAS-NA (Zs = 2.67–3.20, ps ≤ 0.008), supporting discriminant evidence.

#### 3.3.3. Incremental Evidence

Incremental Evidence in Predicting Anxiety. Two hierarchical linear regression models were conducted to examine whether the SST-M significantly predicted^2^ anxiety symptoms (HADS-A and GAD-7) above gender and PANAS-NA. In the HADS-A model, gender did not significantly predict HADS-A in Step 1 (β = 0.018, *p* = 0.88, n.s.). In Step 2, PANAS-NA significantly predicted HADS-A (β = 0.396, *p* < 0.01). In Step 3, the SST-M remained a significant predictor of HADS-A (β = 0.504, *p* < 0.001), explaining an additional 24.1% of the variance (ΔR^2^ = 0.241), while PANAS-NA remained significant (Table 4). In the GAD-7 model, gender did not significantly predict GAD-7 in Step 1 (β = 0.093, *p* = 0.46, n.s.). When PANAS-NA was added in Step 2, it significantly predicted GAD-7 (β = 0.520, *p* < 0.001). In Step 3, the SST-M also significantly predicted GAD-7 (β = 0.406, *p* < 0.001), accounting for an additional 15.1% of the variance (ΔR^2^ = 0.151), while PANAS-NA remained significant (Table 4).

Incremental Evidence in Predicting Depression. Two additional hierarchical linear regression models were used to examine whether the SST-M significantly predicted depressive symptoms (HADS-D and PHQ-9) above gender and PANAS-NA. In Steps 1 and 2, neither gender (β = −0.08, *p* = 0.50, n.s.) nor PANAS-NA (β = 0.141, *p* = 0.27, n.s.) was a significant predictor of HADS-D. In Step 3, gender and PANAS-NA remained non-significant, whereas the SST-M significantly predicted HADS-D (β = 0.562, *p* < 0.001), accounting for an additional 30.3% of the variance (ΔR^2^ = 0.303). In the PHQ-9 model, gender did not significantly predict PHQ-9 (β = 0.089, *p* = 0.49, n.s.) in Step 1. However, in Step 2, PANAS-NA showed a significant, positive effect (β = 0.379, *p* < 0.01). In Step 3, the SST-M significantly predicted PHQ-9 (β = 0.470, *p* < 0.001), accounting for an additional 20.3% of the variance (ΔR^2^ = 0.203), with PANAS-NA remaining significant (Table 5).

## 4. Discussion

The aim of the current study was to examine whether an SST that lacked specific time constraints, cognitive load and negative mood induction was sufficient to find a relationship between IB and current psychopathology symptoms in a university student sample. A concurrent objective was to develop and examine psychometric properties of a modified version of the SST that is considerably easier to implement across larger and broader populations. Consistent with our hypotheses, the SST-M displayed excellent internal consistency reliability. Specifically, we found a very high estimate for categorical omega. However, due to the observed item-level skew and limited variability, this should be interpreted with some caution. Cronbach’s alpha is reported as a more conservative estimate of internal consistency and also suggests excellent internal consistency reliability. Relatedly, items that may reflect more specific interpersonal or appearance-related evaluations showed comparatively lower associations with the total score, suggesting potential heterogeneity in item content. Similarly, our results provided strong evidence based on expected relations with other variables, as indicated by moderate to large correlations between the SST-M and two independent measures of depression, as well as two independent measures of anxiety. In contrast, the SST-M showed a very weak correlation with growth mindset tendencies as well as a weak correlation with negative affect. Specifically, these correlations were significantly weaker than the correlations between SST-M and depression and anxiety, providing discriminant evidence. Incremental evidence in the context of depression and anxiety was also supported. Taken together, our study offers initial support for the intended interpretation of SST-M scores as reflecting IB that predicts current psychopathology symptoms in a university sample.

Notably, our results are comparable to the psychometric properties of the SST reported in previous studies. For example, a meta-analysis by Würtz et al. (2022) reported a pooled internal consistency of *α* = 0.79, with the present study showing a somewhat higher estimate (*α* = 0.89). Similarly, for convergent evidence, Würtz et al. (2022) also reported moderate to high pooled associations between the SST and related constructs (r = 0.46), with the present study yielding estimates in a comparable range (r = 0.478–0.544).

Our results also provide discriminant evidence. IB, negative affect, and growth mindset tendencies are all associated with socio-emotional functioning (Chen et al., 2020; Clark & Watson, 1991; Crawford & Henry, 2004; Everaert et al., 2017; Lei et al., 2024; Hu et al., 2022; Tao et al., 2022). Negative affect has been shown to be associated with depression and anxiety (Clark & Watson, 1991; Crawford & Henry, 2004), and growth mindset has been shown to be associated with resilience, adaptive coping, and improved mental health (Lei et al., 2024; Hu et al., 2022; Tao et al., 2022). The small and non-significant associations between IB and these two constructs suggest that IB is not a general indicator of socio-emotional functioning; rather, it represents a more specific interpretational process.

These psychometric findings are particularly informative when considered alongside prior work examining how task constraints, such as cognitive load, influence the detection of IB. It is important to distinguish between research assessing IB’s role in predicting future depression onset or revealing latent vulnerability, and those, including the current study, that examine its relationship with current symptom severity. Specifically, among individuals at greater risk of depression, longitudinal evidence shows that adding cognitive load can be important for detecting IB. For example, a longitudinal study by Rude et al. (2003) found that IB predicted later onset of major depressive disorder only when the SST was completed under cognitive load. When the SST was administered without cognitive load, it did not predict depression onset. However, in another longitudinal study (Rude et al., 2002) that incorporated a shorter follow-up period (4–6 weeks), researchers found that the SST without cognitive load showed weaker and less consistent, yet still significant associations with depressive symptoms. Watkins and Moulds (2007) reported a similar pattern. Individuals with remitted depression did not differ from never-depressed controls on negative sentence completions when the SST was administered without cognitive load. However, when cognitive load was added, the remitted depression group produced more negative interpretations, similar to currently depressed individuals (see also Martin-Romero & Sanchez-Lopez, 2023). Indeed, negative mood induction has also been proposed as a mechanism for activating latent cognitive vulnerability, such as remitted depression (Beck, 2008; Scher et al., 2005; Lau et al., 2004; Gemar et al., 2001). From this perspective, mood induction procedures could be relevant in designs aimed at detecting hidden vulnerability or prospective risk.

At the same time, the need for cognitive load may depend on the research goal and the sample being studied. Viviani et al. (2018) suggest that SSTs administered without cognitive load may reflect individuals’ spontaneous tendencies in cognition formation rather than effortful executive regulation. If so, in samples where IB is already expressed in relation to current symptom severity, these spontaneous tendencies may be directly observable without the need for additional task constraints. In other words, cognitive load or negative mood induction may be especially useful for revealing hidden vulnerability in remitted or high-risk individuals by reducing the influence of intact executive control. In contrast, the SSTs without cognitive load may capture spontaneous interpretations under low-constraint conditions. Similarly, in a study of university students examining IB in relation to personality traits, the associations between IB and internalizing traits such as negative affectivity and detachment were consistent across SST conditions with and without time limits (Kienhöfer et al., 2024). The SST-M may be particularly suitable for assessing IB in non-clinical or unselected samples, where vulnerability and symptomatology is expressed along a continuum. However, given that the study design does not allow a direct comparison between the traditional SST and the SST-M, our findings do not permit conclusions about whether these measures are interchangeable.

Importantly, our findings on incremental associations suggest that IB is empirically distinguishable from negative affect. Negative affect has been shown to be a significant predictor of depression, independent of IB (Clark & Watson, 1991; Crawford & Henry, 2004). The same is true in our SST-M measure. That is, in our analyses, participants’ level of negative affect significantly predicted one of our depression measures (PHQ-9), with a moderate effect size. Nonetheless, IB (measured by the SST-M) significantly predicted depression above and beyond participants’ negative affect, with a moderate-to-large incremental effect. Broadly, these findings may illustrate how depression can reflect multiple processes beyond IB.

Similarly, the SST-M accounted for unique variance in anxiety symptoms above and beyond gender and negative affect, with a moderate-to-large effect size, providing further support for the validity evidence. Indeed, as mentioned above, the SST-M in our study did not significantly correlate with negative affect, suggesting that IB and negative affect are distinct constructs. These findings contribute to ongoing discussion regarding whether IB reflects variance beyond general negative affect or mood-congruent responding (for a review, see Everaert et al., 2017). However, given the cross-sectional nature of the study design, these interpretations should be made with caution. Importantly, it should be noted that the current findings do not equate to incremental utility in diagnosis, prognosis, or prediction over time.

In addition, it is noteworthy that negative affect in our study was significantly associated with one of our depression measures, the PHQ-9 (Kroenke et al., 2001), but not the other, the HADS-D (Zigmond & Snaith, 1983), despite showing a strong association with one another (r = 0.675) and demonstrating similar patterns of association with other study variables (e.g., anxiety and IB). Indeed, one study found that seven widely used measures of depressive symptoms, although not including HADS-D and PHQ-9, share only about 41% overlap in symptom content and may therefore produce different results within the same sample (Fried, 2017). For instance, the PHQ-9 maps onto DSM-5 criteria for major depressive disorder, whereas the HADS-D does not and specifically excludes somatic symptoms of depression. Thus, one possible explanation is that the negative affect in our sample was more strongly related to depressive features captured by the PHQ-9, including somatic or broader DSM-based symptoms, than to the symptom profile assessed by the HADS-D. However, future research is needed to test this possibility directly (e.g., examination of symptom-level associations).

Interestingly, we found that women demonstrated significantly higher SST-M scores than men. Prior findings on gender differences in IB have been mixed. For example, Krahe et al. (2022) did not observe gender differences in the SST performance, and Berna et al. (2011) similarly reported no gender differences using a non-SST IB task. In contrast, Barroso et al. (2025) reported significant gender differences in IB in a mathematics context using another non-SST IB paradigm. Given the small sample size in the current study and the over-representation of women in our sample, the findings regarding gender should be interpreted cautiously. Further research is needed to clarify whether gender differences in IB emerge, and if so, why and in which contexts.

This study has limitations that warrant discussion. First, in addition to the imbalanced gender representation, the use of a university student sample may further limit the generalizability. While a sizable proportion of participants reported elevated symptoms of anxiety, with 33.3% to 39.4% meeting the established clinical cut-off for independent measures of anxiety, we acknowledge that the sample consisted of psychology students from a single Canadian university, most of whom were women. Second, although sensitivity analyses indicated adequate power to detect medium-sized effects, the sample size may have limited sensitivity to smaller effects and may have resulted in unstable parameter estimates, warranting caution and the need for replication. Third, while this study offers validity evidence based on internal consistency reliability and relationships with other variables, it lacks evidence related to response processes and test–retest reliability. Fourth, as mentioned above, our current study design does not allow us to draw conclusions on whether the performance and utility of SST-M is comparable to the traditional SST. Thus, current results should be interpreted as initial support for the SST-M to detect individual differences in IB and predict symptoms of current psychopathology. We are not making any claims about its performance relative to the traditional SST.

With these aforementioned limitations in mind, future research should replicate these findings in larger and more diverse samples. Importantly, future research adopting a longitudinal design would establish whether the SST-M can prospectively predict depressive and anxiety symptoms, particularly among at-risk individuals. In addition, incorporating a well-established measure of IB as another source of convergent validity evidence would be beneficial. Similarly, while the present study focused on broader socio-emotional constructs, it may be informative to evaluate discriminant validity with respect to other negatively valenced cognitions that are more proximal to IB. For example, prior work suggests that antisocial cognitions, although negatively valenced, are distinct from depressive cognitive styles (e.g., Rabl et al., 2024). Future research should test whether the SST-M captures an IB that is also distinct from other domains of maladaptive cognition. Furthermore, comparing the SST-M with traditional SST paradigms that include time limits, concurrent cognitive load, and mood induction may also offer better insight into the conditions under which these procedural features contribute to the assessment of IB. Lastly, in line with the emphasis on the need to establish temporal stability of task-based measures (Parsons et al., 2019), test–retest reliability should be examined in future research.

## 5. Conclusions

The Scrambled Sentences Task (SST) is a widely used experimental paradigm for measuring negative interpretation bias (IB). We examined the validity evidence and reliability of scores from a modified version of the SST (SST-M) that lacks time limits, cognitive load, and negative mood induction. Our study offers several important contributions. Our results reveal that IB can be reliably detected in a self-administered, online format without time limits, concurrent cognitive load, or negative mood induction in a university student sample using a cross-sectional design. In other words, these constraints may not be required in all research contexts to assess meaningful individual differences in IB that are associated with psychological functioning. In addition to this theoretical contribution to the field, the SST-M provides several practical and research-forward benefits for the field. The SST-M represents an important methodological advance, offering an easy-to-use method that eliminates many barriers associated with traditional lab-based SST paradigms. By substantially lowering barriers to data collection, it offers potential for faster study deployment, large-scale screening, longitudinal follow-ups, and cross-site replication, supporting cumulative progress in the field. Its potential for increased accessibility will hopefully facilitate recruitment of larger and more diverse samples, enhancing statistical power and improving the generalizability of findings to populations historically underrepresented in laboratory-based research. Further research in more diverse and clinical samples is required to confirm these benefits. Collectively, these advantages position the SST-M as a user-friendly and inclusive tool with the potential to meaningfully advance research.

## Figures and Tables

**Table 1:** Participant Demographic Characteristics (*N* = 66).

	Total Sample (*N* = 66)
	*M*(*SD*) or %(*n*)
Age	20.58 (1.93)
Gender	
Women	80.3% (53)
Men	19.7% (13)
Gender Non-Binary	0 (0)
Ethnicity	
Asian	57.6% (38)
White	28.8% (19)
Latinx	3% (2)
Preferred to self-describe	10.6% (7)
Education	
High school or equivalent	57.6% (38)
Some post-secondary	33.3% (22)
Associate’s degree	5% (3)
Bachelor’s degree/RN	5% (3)

Note. RN = Registered nurse; Some post-secondary: Participants who have completed some coursework in post-secondary education but have not obtained a degree, diploma, or certificate are included in this category.

**Table 2:** Descriptive Statistics.

	Mean	SD	Min	Max	Skewness	Kurtosis
SST-M	0.25	0.20	0	0.70	0.91	−0.13
HADS-A	9.64	4.56	0	20.00	0.02	−0.57
GAD-7	7.32	5.78	0	21.00	0.74	−0.49
HADS-D	6.33	4.10	0	16.00	0.52	−0.53
PHQ-9	9.77	6.27	0	25.00	0.53	−0.43
ITIS	4.21	1.09	1.67	6.00	−0.36	−0.52
NA	24.50	8.46	10	47.00	0.17	−0.83

Note. SST-M = Scrambled Sentences Task-Modified; HADS-A = Hospital Anxiety and Depression Scale–Anxiety subscale (Zigmond & Snaith, 1983); GAD-7 = Generalized Anxiety Disorder-7 scale (Spitzer et al., 2006); HADS-D = Hospital Anxiety and Depression Scale–Depression subscale (Zigmond & Snaith, 1983); PHQ-9 = Patient Health Questionnaire-9 for depressive symptoms (Kroenke et al., 2001); ITIS = Implicit Theory of Intelligence Scale (Dweck et al., 1995); NA = Negative Affect subscale of the Positive and Negative Affect Schedule (D. Watson et al., 1988).

**Table 3:** Zero-order Bivariate Correlations.

	1. SST-M	2. HADS-A	3. GAD-7	4. HADS-D	5. PHQ-9	6. ITIS	7. NA
1	–	0.544 ***	0.478 ***	0.540 ***	0.515 ***	−0.080	0.147
2	–	–	0.800 ***	0.700 ***	0.741 ***	−0.061	0.396 **
3	–	–	–	0.565 ***	820 ***	−0.017	0.520 ***
4	–	–	–	–	0.675 ***	−0.049	0.139
5	–	–	–	–	–	0.089	0.377 **
6	–	–	–	–	–	–	−0.094
7	–	–	–	–	–	–	–

Note. ** *p* < 0.001. *** *p* < 0.0001. SST-M = Scrambled Sentences Task-Modified; HADS-A = Hospital Anxiety and Depression Scale–Anxiety subscale (Zigmond & Snaith, 1983); GAD-7 = Generalized Anxiety Disorder-7 scale (Spitzer et al., 2006); HADS-D = Hospital Anxiety and Depression Scale–Depression subscale (Zigmond & Snaith, 1983); PHQ-9 = Patient Health Questionnaire-9 for depressive symptoms (Kroenke et al., 2001); ITIS = Implicit Theory of Intelligence Scale (Dweck et al., 1995); NA = Negative Affect subscale of the Positive and Negative Affect Schedule (D. Watson et al., 1988).

**Table 4:** Hierarchical Regression Models of SST-M and Anxiety.

	Model 1 (HADS-A)			Model 2 (GAD-7)		
	Step1	Step 2	Step 3	Step 1	Step 2	Step 3
	Standardized Coefficient					
Gender	0.018 [−0.23, 0.27]	0.007 [−0.23, 0.24]	−0.092 [−0.29, 0.11]	0.093 [−0.16, 0.34]	0.078 [−0.14, 0.29]	−0.002 [−0.20, 0.20]
PANAS-NA		0.396 ** [0.16, 0.63]	0.324 ** [0.12, 0.53]		0.520 *** [0.30, 0.74]	0.463 *** [0.26, 0.66]
SST-M			0.504 *** [0.30, 0.71]			0.406 *** [0.21, 0.61]
F Statistic	F(1, 62) = 0.213	F(2, 61) = 5.686	F(3, 60) = 13.39	F(1, 62) = 0.548	F(2, 61) = 11.63	F(3, 60) = 15.2
Model R^2^	0.0003	0.157	0.401	0.009	0.276	0.432
Model Adjusted R^2^	−0.016	0.130	0.371	−0.007	0.252	0.403
Change in R^2^		0.146 ***	0.241 ***		0.259 ***	0.151 ***

Note. 95% confidence intervals are reported in brackets. ** *p* < 0.001. *** *p* < 0.0001. PANAS-NA Positive and negative affect schedule—Negative affect (D. Watson et al., 1988); SST-M = Scrambled Sentences Task-Modified; HADS-A = Hospital Anxiety and Depression Scale–Anxiety subscale (Zigmond & Snaith, 1983); GAD-7 = Generalized Anxiety Disorder-7 scale (Spitzer et al., 2006).

**Table 5:** Hierarchical Regression Models of SST-M and Depression.

	Model 1 (HADS-D)			Model 2 (PHQ-9)		
	Step1	Step 2	Step 3	Step 1	Step 2	Step 3
	Standardized Coefficient					
Gender	−0.08 [−0.33, 0.17]	−0.09 [−0.34, 0.16]	−0.198 [−0.41, 0.01]	0.089 [−0.16, 0.34]	0.077 [−0.15, 0.31]	−0.014 [−0.23, 0.20]
PANAS-NA		0.141 [−0.11, 0.39]	0.062 [−0.15, 0.27]		0.379 ** [0.14, 0.62]	0.312 ** [0.10, 0.52]
SST-M			0.562 *** [0.35, 0.78]			0.470 *** [0.26, 0.68]
F Statistic	F(1, 62) = 0.456	F(2, 61) = 0.860	F(3, 60) = 9.921	F(1, 62) = 0.492	F(2, 61) = 5.296	F(3, 60) = 11.01
Model R^2^	0.007	0.027	0.332	0.008	0.148	0.355
Model Adjusted R^2^	−0.009	−0.004	0.298	−0.008	0.12	0.323
Change in R^2^		0.004	0.303 ***		0.112 ***	0.203 ***

Note. 95% confidence intervals are reported in brackets. ** *p* < 0.001. *** *p* < 0.0001. PANAS-NA Positive and negative affect schedule—Negative affect (D. Watson et al., 1988); SST-M = Scrambled Sentences Task-Modified; HADS-D = Hospital Anxiety and Depression Scale–Depression subscale (Zigmond & Snaith, 1983); PHQ-9 = Patient Health Questionnaire-9 for depressive symptoms (Kroenke et al., 2001).

## Data Availability

The original data presented in the study are openly available in Open Science Foundation at https://doi.org/10.17605/OSF.IO/7MHCN.

## Notes

1 Participants’ self-described identities included: Mixed (*n* = 2; White & Asian [*n* = 1]), South Asian/Indian (*n* = 3), Middle Eastern (*n* = 1), African (*n* = 1). While broader categories (e.g., Asian, Black) were provided as response options, identities are reported here as self-described to reflect participants’ preferred wording.

2 Note that the term “predict” is used in a statistical rather than causal sense. Given the cross-sectional nature of the data, no causal inferences are intended.

## Supplementary Materials

The following supporting information can be downloaded at: https://www.mdpi.com/article/10.3390/bs16050705/s1, Table S1: Demographic and Clinical Characteristics by Inclusion Status, Table S2: Item-level statistics for SST-M trials.

## Abbreviations

The following abbreviations are used in this manuscript:

IB	Negative interpretation bias
SST-M	Scrambled sentences task—Modified
HADS-A	Hospital anxiety and depression scale—Anxiety (Zigmond & Snaith, 1983)
HADS-D	Hospital anxiety and depression scale—Depression (Zigmond & Snaith, 1983)
GAD-7	Generalized anxiety disorder-7 (Spitzer et al., 2006)
PHQ-9	Patient health questionnaire-9 (Kroenke et al., 2001)
ITIS	Implicit theory of intelligence scale (Dweck et al., 1995)
PANAS-NA	Positive and negative affect schedule—Negative affect (D. Watson et al., 1988)
