# Supplementary material for: Negativity Bias in Depression and Anxiety: Examining the Psychometric Properties of a Modified Scrambled Sentences Task to Measure Interpretation Bias
Source: Behav Sci (Basel). 2026 May 5;16(5):705. doi: 10.3390/bs16050705 (PMC13203341; doi:10.3390/bs16050705)
Supplement: Supplementary file 1 [file behavsci-16-00705-s001.zip › behavsci-4243993-supplementary.pdf]

Supplementary Materials

Table S1. Demographic and Clinical Characteristics by Inclusion Status

	**Participants Included (*N*=66)**	**Participants Excluded (*N*=9)**
	M(*SD*) or %(*n*)	
Age	20.58 (1.93)	20.00 (1.22)
Gender		
Women	80.3% (53)	88.8% (8)
Men	19.7% (13)	11.1% (1)
Gender Non-Binary	0 (0)	0 (0)
Ethnicity		
Asian	57.6% (38)	55.5% (5)
White	28.8% (19)	22.2% (2)
Latinx	3% (2)	0 (0)
Preferred to self-describe	10.6% (7)	22.2% (2)
Education		
High school or equivalent	57.6% (38)	66.6% (6)
Some post-secondary	33.3% (22)	22.2% (2)
Associate's degree	5% (3)	0 (0)
Bachelor's degree/RN	5% (3)	11.1% (1)
Symptom Measures		
HADS-A	9.64 (4.56)	11.44(2.35)
GAD-7	7.32 (5.78)	7.89 (6.31)
HADS-D	6.33 (4.10)	9.22 (2.59)
PHQ-9	9.77 (6.27)	10.11 (6.72)
ITIS	4.21 (1.09)	4.51 (1.30)
NA	24.50 (8.46)	24.11 (7.11)

Note. RN = Registered nurse; Some post-secondary: Participants who have completed some coursework in post-secondary education but have not obtained a degree, diploma, or certificate are included in this category. HADS-A = Hospital Anxiety and Depression Scale–Anxiety subscale (Zigmond & Snaith, 1983); GAD-7 = Generalized Anxiety Disorder-7 scale (Spitzer et al., 2006); HADS-D = Hospital Anxiety and Depression Scale–Depression subscale (Zigmond & Snaith, 1983); PHQ-9 = Patient Health Questionnaire-9 for depressive symptoms (Kroenke et al., 2001); ITIS = Implicit Theory of Intelligence Scale (Dweck et al., 1995); NA = Negative Affect subscale of the Positive and Negative Affect Schedule (Watson et al., 1988).

Table S2. Item-level statistics for SST-M trials

Item	Mean (p)	Variance	Corrected item–total r
1. my on I mistakes achievements focus	0.44	0.251	0.474
2. am I equal others to inferior	0.91	0.083	0.501
3. worthwhile person I worthless am a	0.85	0.132	0.516
4. I am generally success a failure	0.86	0.120	0.606
**5. disgusting attractive appearance my physical is**	**0.82**	**0.153**	**0.193**
6. am unacceptable acceptable person I an	0.79	0.170	0.567
7. I confident disappointed in am myself	0.77	0.178	0.647
8. I born was winner a loser	0.88	0.108	0.315
9. capable generally am quite I incapable	0.95	0.044	0.438
10. laughing feel crying like I often	0.56	0.250	0.445
11. happy make things me most unhappy	0.90	0.092	0.351
12. are decisions make easy difficult to	0.48	0.254	0.539
13. I feel very calm usually nervous	0.72	0.203	0.524
14. succeed once will I fail more	0.82	0.152	0.626
15. achieve will I abandon goals my	0.85	0.131	0.427
16. joyful expect to I miserable be	0.85	0.131	0.737
17. about I think worry the future	0.54	0.252	0.339
18. people to me exclude like include	0.67	0.226	0.553

19. other people usually me like dislike	0.86	0.120	0.315
**20. respect ignore opinions my friends my**	**0.89**	**0.099**	**-0.022**
21. helped lost have I friends my	0.76	0.186	0.364
**22. again support others criticize me will**	**0.80**	**0.164**	**0.204**
23. bad to things seem good happen	0.55	0.252	0.676
24. am I my improving life ruining	0.94	0.058	0.517
25. my interesting boring is generally life	0.61	0.242	0.409
26. possible impossible achieve happiness is to	0.84	0.134	0.472
27. failed at life I succeeded have	0.70	0.214	0.476
28. positive my are negative memories mostly	0.78	0.172	0.509
29. optimistic things am about pessimistic I	0.82	0.151	0.545
30. full life of is challenges opportunities	0.35	0.232	0.501

Note. Responses were coded as 1 = positive and 0 = negative, item means represent the proportion of participants endorsing the positive interpretation. Bold statements reflect items with corrected-item total correlations <30.
